# Kaposiform hemangioendothelioma complicated by Kasabach–Merritt phenomenon in an infant girl

**DOI:** 10.1002/ccr3.7859

**Published:** 2023-09-13

**Authors:** Louise Helligsø, Torben Stamm Mikkelsen, Anne‐Mette Hvas

**Affiliations:** ^1^ Present address: Department of Ophtalmology Aarhus University Hospital Aarhus Denmark; ^2^ Present address: Department of Pediatrics Aarhus University Hospital Aarhus Denmark; ^3^ Present address: Faculty of Health Aarhus University Aarhus Denmark

**Keywords:** Kaposiform hemangioendothelioma, Kasabach–Merritt syndrome, pericardial effusion, Sirolimus, thrombocytopenia

## Abstract

**Key Clinical Message:**

We report a successful treatment course of an infant with mediastinal Kaposiform hemangioendothelioma. As the current complex of diseases is rare and calls for highly specialized treatment, large prospective studies are lacking. This case provides an example of balanced treatment complicated by Kasabach–Merritt phenomenon, life‐threatening infections, and pericardial effusion.

**Abstract:**

Kaposiform hemangioendothelioma (KHE) and tufted angioma are vascular benign tumors that can be associated with the rare condition Kasabach–Merritt Phenomenon (KMP). KMP is characterized by consumption coagulopathy with severe thrombocytopenia, hypofibrinogenemia, and elevated D‐dimer. We here report successful treatment of a female infant with a mediastinal KHE where treatment was complicated by KMP, life‐threatening infections, and pericardial effusion. Due to the absence of randomized clinical trials, there is currently no standardized treatment protocol available for KHE. In our case, the infant was treated successfully with prednisolone, vincristine, and sirolimus.

## Graphical abstract

Above is depicted a one week old infant girl with Kaposiform hemangioendothelioma (KHE) at the peak of Kasabach‐Merritt phenomenon (KMP), with edema, bluish discoloration of the skin, and a firm swelling at the throat. KMP is characterized by consumption coagulopathy with severe thrombocytopenia, hypofibrinogenemia, and elevated D‐dimer. We here report successful treatment of a female infant with a mediastinal KHE where treatment was complicated by KMP, life‐threatening infections, and pericardial effusion.

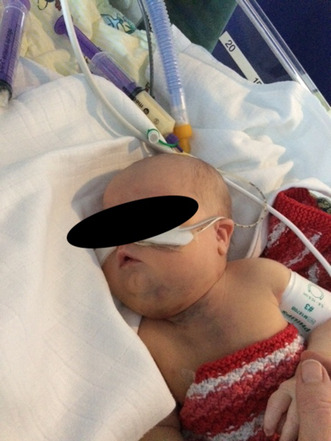

## 1 INTRODUCTION

Kaposiform hemangioendothelioma (KHE) and the rarer tufted angioma are vascular tumors that can be associated with the life‐threatening condition Kasabach–Merritt phenomenon (KMP). KHE is a benign tumor that can cause complications as it grows, including infiltration and compression of nearby organs.^1^ The KHE tumors can be located superficially in the skin and in deeper regions of the body. In infants, KHE are most often located at the extremities, trunk, head/neck, whereas only about 5% are located in the mediastinum.^1^ If the tumor is located on the throat or neck, congenital salivary gland tumor is a differential diagnosis to KHE.^2^, ^3^ Infants and patients with KHE located in the mediastinum often experience KMP as a complication.^1^ KMP, first described by Kasabach H. Merritt in 1940,^4^ is characterized by severe consumption coagulopathy with thrombocytopenia, hypofibrinogenemia, and elevated D‐dimer. KMP may also be complicated by severe anemia due to blood sequestration and bleeding within the tumor.^4^ In patients with KHE complicated by KMP, the mortality reaches 30%.^1^ In a systematic review conducted by Schmid et al., it was found that 79% of infants with KHE also exhibited KMP, indicating a strong association between the two conditions. Similarly, 47% of children aged 1–5 years, 43% of those aged 6–12 years, and only 10% of patients aged 13–21 years presented with KMP in conjunction with KHE.^1^

Treatment of KHE with KMP is challenging due to the potential for platelet concentrate transfusions to promote tumor growth, exacerbating the consumption coagulopathy. Weekly infusions with vincristine in combination with daily administration of prednisolone often cause tumor regression and reduce the KMP.^5^, ^6^ Sirolimus can be used exclusively or in conjunction with vincristine and prednisolone.^1^, ^7^

We here report a complicated, but successful, treatment course of an infant with KHE complicated by KMP, pericardial effusion, and life‐threatening infections.

## 2 CASE

**FIGURE 1:**
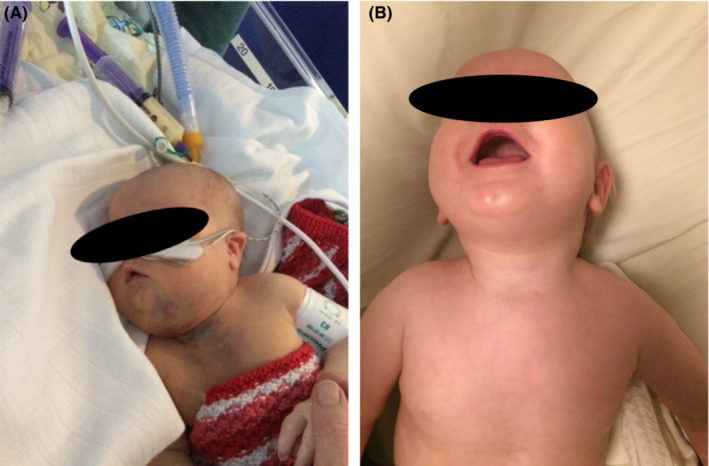
Kaposiform hemangioendothelioma with Kasabach‐Merritt phenomenon before and after treatment. (A) At the peak of Kasabach‐Merritt phenomenon (1 week old), with edema, bluish discoloration of the skin, and a firm swelling at the throat. (B) After 1 year of therapy with sirolimus and prednisolone, complete resolution of the cutaneous components was achieved.

At birth, a female infant was transferred to our neonatal ward due to persistent oxygen needs, thrombocytopenia (platelet count 26 × 10^9^/L), and a bluish‐purple discoloration at the anterior part of the neck that was thought to be a hematoma. The pregnancy had been without complications and the infant was born vaginally at term. Clinical inspection of the new‐born revealed edema at the anterior neck and upper thorax. A bluish discoloration was observed at the firm swelling on the throat. On the skin, discrete superficial vascular abnormalities were identified on the upper thorax. In addition, petechiae were present on the truncus, hips, and nates (Figure 1A). Ultrasound of the throat showed diffuse swollen soft tissue and a computer tomography scan showed small condensations in the subcutaneous fat tissue located antero‐apical to the thorax and the anterior part of the throat. No maternal antiplatelet antibodies were found. The platelet count showed an initial increase after each platelet concentrate transfusion. However, with an increasing number of transfusions, the platelet count started to decrease at a faster rate, and reached lower levels. (Table 1). After administration of platelet concentrates, the firm swelling on the throat expanded. A consumption coagulation developed as indicated by thrombocytopenia shortly after birth with a continued need for platelet concentrate transfusion, reduced fibrinogen from day two, and prolonged activated partial thromboplastin time at Day 7.

**TABLE 1:**
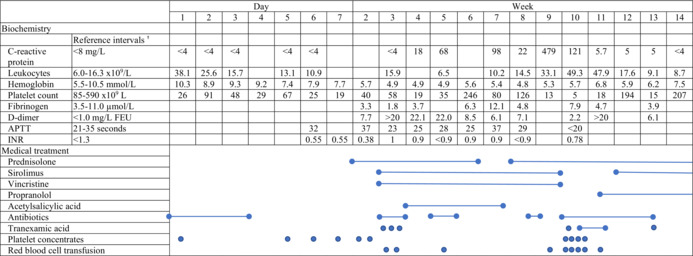
Biochemistry and medical treatment of an infant from 0 to 14 weeks with Kaposiform hemangioendothelioma and Kasabach–Merritt phenomenon.

Abbreviations: APTT, activated partial thromboplastin time; INR: international normalized ratio.

^a^ Reference intervals are provided according to the patient's age.

**FIGURE 2:**
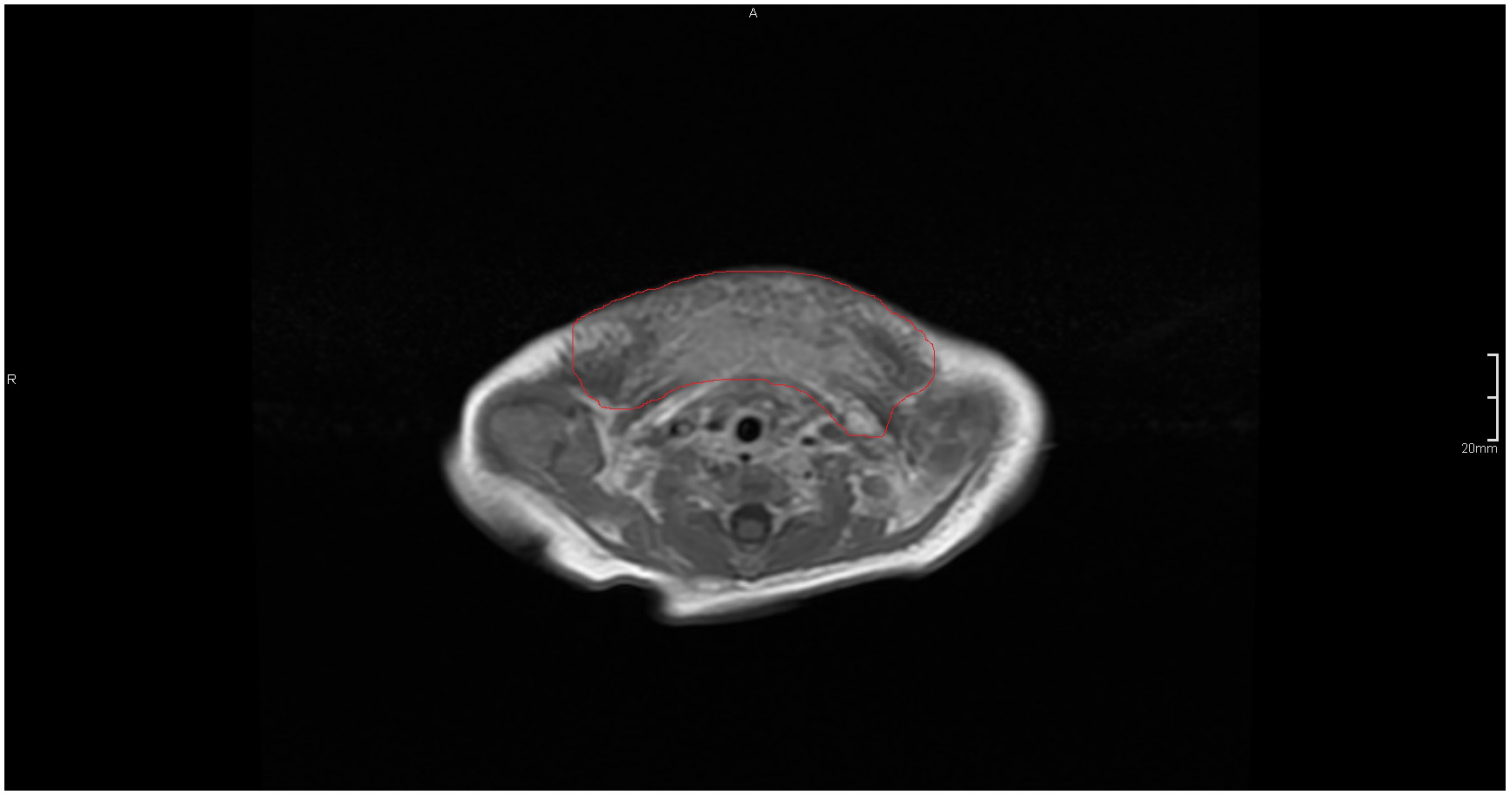
Magnetic resonance imaging: T1 with gadolinium contrast depicts the tumor tissue in white.

**FIGURE 3:**
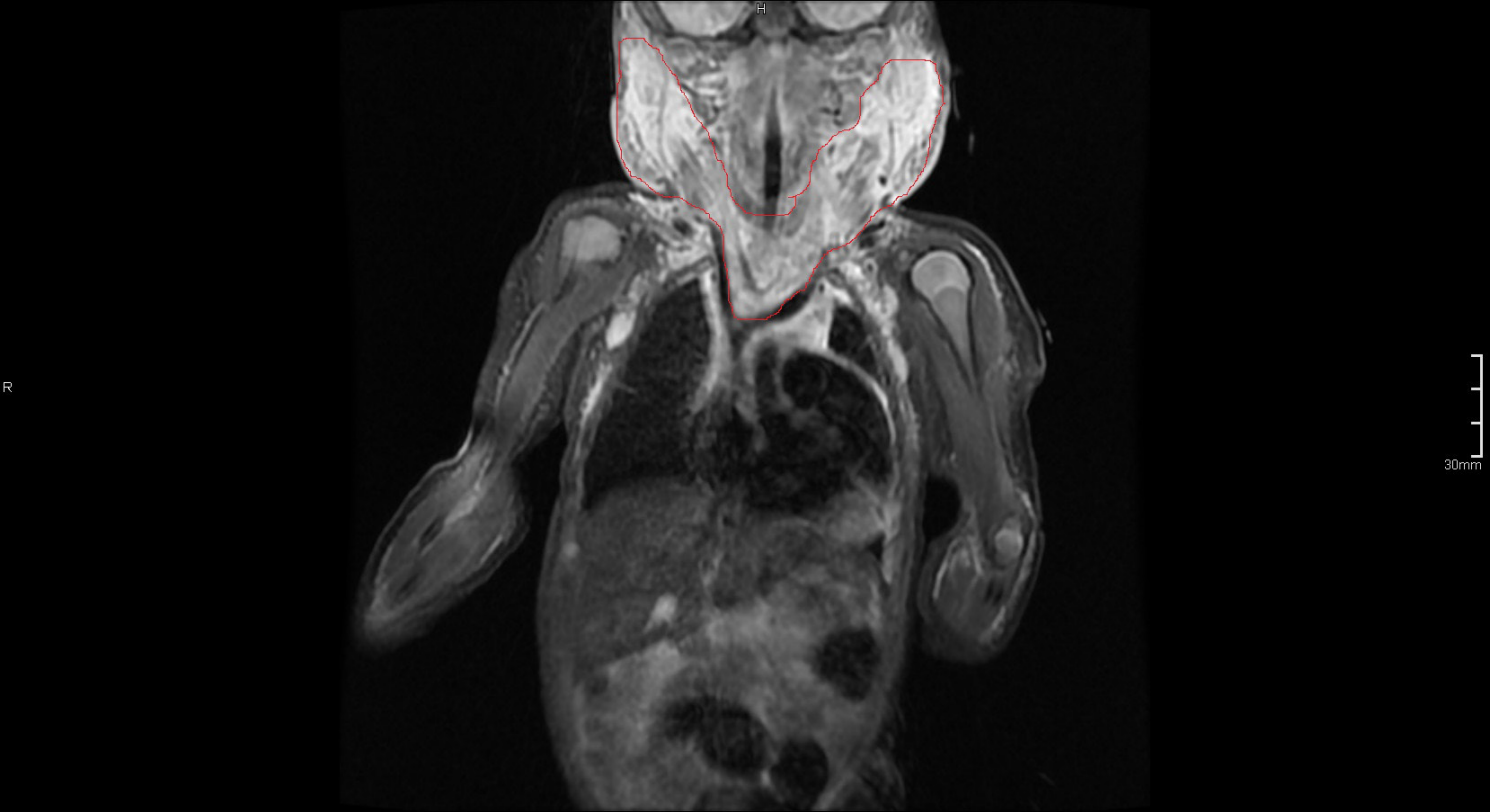
Magnetic resonance imaging: Short T1 inversion recovery (STIR) sequence where the tumor edema is depicted in white.

Eleven days after birth, the infant was intubated due to respiratory insufficiency and a diagnostic magnetic resonance imaging (MRI) was performed. Gadolinium contrast‐enhanced MRI revealed diffuse enhancement in the deeper mediastinal structures without the presence of a sharply demarcated tumor or hematoma (Figure 2). Short T1 inversion recovery (STIR) MRI sequence showed diffuse changes on both sides of the neck and superior mediastinum, suggesting edema in the subcutaneous structures (Figure 3). The diagnosis of KHE complicated by KMP was made based on clinical observations, biochemistry, and MRI. No biopsy was obtained for confirmation at any given time because of high bleeding risk and respiratory insufficiency. Prednisolone treatment (2 mg/kg per day) was initiated after the MRI, and the first dose with vincristine (0.05 mg/kg per week) was given 3 days later (Day 15) after placement of a central venous catheter (CVC). At Day 17, oral sirolimus (0.8 mg/m^2^ twice daily, target: 5–10 μg/L) was added to the treatment. This decision was made due to the critical clinical situation, including the need for invasive respiratory support, and persistent signs of consumption coagulopathy.

A pericardial effusion, diagnosed on day one, increased gradually in volume (20 mm), resulting in a “swinging heart”. At Day 20, when the platelet count was 39 × 10^9^/L and plasma fibrinogen 2.0 μmol/L, a pericardiocentesis was performed after administration of intra venous (IV) fibrinogen concentrate at a dosage of 30 μg/kg, as well as a platelet concentrate transfusion. Laboratory analysis of the pericardial fluid revealed a high concentration of triglyceride; and after introducing a diet primarily containing medium‐chain triglycerides, the pericardial exudate disappeared. To further inhibit the platelet activity and prevent thrombus formation in the tumor, oral acetylsalicylic acid at a dosage of 2 mg/kg/day was initiated after the platelet transfusion administered in relation to the pericardiocentesis. Attempts at reducing prednisolone resulted in thrombocytopenia and secondary tumor growth at Week 7 (Table 1).

Ten weeks old, when the infant had been without respiratory support for 3 days, she suffered a CVC infection with Klebsiella pneumoniae. During Week 10, the platelet count dropped from 109 × 10^9^/L to 55 × 10^9^/L during a single day. Despite receiving platelet concentrates during intubation, she developed severe respiratory distress and blood was aspirated from the respiratory tract. Due to a suspected lung bleeding, she received two additional platelet transfusions during the following 2 days. This resulted in tumor growth and a nadir platelet count at 5 × 10^8^/L. Treatment with acetylsalicylic was discontinued and treatment with IV tranexamic acid (TXA) (10 mg/kg) was initiated.

Because of the infection, the CVC had to be removed, thereby discontinuing vincristine treatment.

To prevent consumption coagulopathy or further tumor growth, it was decided to administer platelet transfusions only in case of severe bleeding and to replace fibrinogen at plasma fibrinogen concentrations <2.5 μmol/L. Transfusion with red blood cells was indicated only if hemoglobin was below 4.5 mmol/L.

Fourteen weeks old, the infant was discharged to the outpatient clinic with prednisolone, sirolimus, and sulfametoxazol with trimetoprim (prophylaxis to Pneumocystis jiroveci). Prednisolone was weaned gradually and discontinued when she was 4 months old. Due to iatrogenic adrenal insufficiency, she had to be treated with oral hydrocortisone until she was 12 months old.

Plasma sirolimus concentration was measured once a month, and the median concentration was 6.4 μg/L. The tumor gradually decreased in size and could not be visualized or palpated when the child was 1 year old (Figure 1B). Childhood vaccinations (at three, six, and 12 months) and an acute infection with respiratory syncytial virus at 14 months did not cause thrombocytopenia or tumor growth. Sirolimus was continued for 2 years and then tapered very slowly over a period of 4 months.

The infant girl, now 4 years and 4 months old, is without recurrence disease 21 months after completing treatment with sirolimus.

## 3 DISCUSSION

A well‐functioning CVC is vital for vincristine administration. However, the occurrence of repeated infections in the CVC, combined with KMP, posed a clinical treatment dilemma. This eventually led to life‐threatening thrombocytopenia and necessitated removal of the CVC. A retrospective study with 10 cases reported by Liu et al. showed that a cocktail therapy is preferable to reduce drug resistance in monotherapy with prednisolone, sirolimus, or vincristine.^8^ In the study by Liu et al., the recommended dosage of vincristine was four cycles (0.05 mg/kg/week). However in our case, the infant girl received seven cycles of treatment with vincristine.

Contrary to this, a meta‐analysis by Peng et al. from 2019 compared the effectiveness of vincristine and sirolimus treatment in regard to tumor shrinkage and/or normalizing platelet count in KHE with or without KMP.^9^ This study revealed that sirolimus had a higher odds ratio (OR) of 0.91 than vincristine with an OR of 0.72. The study also showed that both vincristine and sirolimus led to a normalized platelet count in KHE with KMP, with an OR of 0.82 for vincristine and 0.94 for sirolimus. This suggests that vincristine may not be crucial in the treatment of KMP.

A noteworthy contribution to the discussion of optimized treatment for KHE with KMP is the recent study by Harber et al. from 2021. This study suggests lower doses of sirolimus (0.2 mg/m^2^ every 24–48 h) and a lower therapeutic target (4–6 μg/L) in infants (<1 year) to avoid adverse and potentially fatal side effects. The hypothesis is based on the maturation of liver enzymes (CYP3A4) in infants, which reach adult levels by 1 year old.^10^

Prednisolone was administered to prevent severe thrombocytopenia; so, the dose in our patient was gradually tapered over time. The medication continued until the infant reached 4 months of age. This observation was substantiated in a randomized clinical trial (RCT) by Ji et al. later in 2020.^11^ In this study, the combination therapy of sirolimus and prednisolone was compared with sirolimus monotherapy. The results showed that the combination therapy group had a higher percentage (94.6%) of patients with a durable platelet response after Week 4, compared with only 66.7% in the monotherapy group. When platelet count was ≥100 × 10^9^/L for at least 1 week, the recommendation in the RCT was to attempt slow tapering of prednisolone. Our patient needed prednisolone treatment for over 10 weeks before gradually tapering the drug. This finding underlines the importance of including prednisolone as a mandatory component in the treatment of KHE with KMP.

Pericardial effusion requiring treatment with drainage is a rare complication to KHE.^12^, ^13^ The pericardial effusion of chylous content resolved when the infant started a diet primarily containing medium‐chain triglycerides. However, it remains unclear whether regression of pericardial effusion was due to the changed diet or the shrinking of the mediastinal tumor.

Microthrombi degradation is essential for tumor shrinkage, but it is inhibited by TXA. Despite our initial reluctance, we had to administer TXA due to the severity of aggressive thrombocytopenia and bleeding.

Due to the rarity of the current complex of diseases and the need for highly specialized treatment, large prospective studies are lacking. In conclusion, the present case provides an example of a well‐balanced treatment approach in an infant with KHE complicated by KMP, pericardial effusion and life‐threatening infections. It can provide valuable insights and guidance for specialists in the field of hematology.

## AUTHOR CONTRIBUTIONS

**Louise Helligsø:** Writing – original draft; writing – review and editing. **Torben Stamm Mikkelsen:** Writing – review and editing. **Anne‐Mette Hvas:** Writing – review and editing.

## FUNDING INFORMATION

There was no source of funding for this study.

## CONFLICT OF INTEREST STATEMENT

The authors declare no conflicts of interest.

## CONSENT

Written informed consent was obtained from the parents to publish this report in accordance with the journal's patient consent policy.

## Data Availability

Author elects to not share data.
